# An Ultraviolet-Lithography-Assisted Sintering Method for Glass Microlens Array Fabrication

**DOI:** 10.3390/mi14112055

**Published:** 2023-11-02

**Authors:** Fangyuan Zuo, Shenghua Ma, Wei Zhao, Chenqian Yang, Ziyu Li, Chen Zhang, Jintao Bai

**Affiliations:** 1 State Key Laboratory of Photon-Technology in Western China Energy, Xi’an 710127, China; zuofy@stumail.nwu.edu.cn (F.Z.); zwbayern@nwu.edu.cn (W.Z.); li_ziyu@stumail.nwu.edu.cn (Z.L.); 2 International Collaborative Center on Photoelectric Technology and Nano Functional Materials, Xi’an 710127, China; mashenghua@nwu.edu.cn (S.M.); yangchenqian@stumail.nwu.edu.cn (C.Y.); 3 Institute of Photonics & Photon Technology, Northwest University, Xi’an 710127, China; 4 Key Laboratory of Optoelectronics Technology in Shaanxi Province, Xi’an 710127, China

**Keywords:** convex microlens arrays, sintering, ultraviolet lithography, glass particle

## Abstract

Glass microlens arrays (MLAs) have tremendous prospects in the fields of optical communication, sensing and high-sensitivity imaging for their excellent optical properties, high mechanical robustness and physicochemical stability. So far, glass MLAs are primarily fabricated using femtosecond laser modification assisted etching, in which the preparation procedure is time-consuming, with each concave-shaped microlens being processed using a femtosecond laser point by point. In this paper, a new method is proposed for implementing large-scale glass MLAs using glass particle sintering with the assistance of ultraviolet (UV) lithography. The glass particles are dispersed into the photoresist at first, and then immobilized as large-scaled micropillar arrays on quartz glass substrate using UV lithographing. Subsequently, the solidified photoresist is debinded and the glass particles are melted by means of sintering. By controlling the sintering conditions, the convex microlens will be self-assembled, attributed to the surface tension of the molten glass particles. Finally, MLAs with different focal lengths (0.12 to 0.2 mm) are successfully fabricated by utilizing different lithography masks. Meanwhile, we also present the optimization of the sintering parameter for eliminating the bubbles in the microlenses. The main factors that affect the focal length of the microlens and the image performance of the MLAs have been studied in detail.

## 1. Introduction

Microlens arrays (MLAs) are one of the essential micro-optical components. For the merits of their light weight, small size and easy integration, MLAs have been widely applied in organic light emitting diodes, 3D displays, light field cameras, solar cells, etc. [1,2,3,4,5].

Generally, MLAs are made of polymers and glass. The fabrication of polymeric MLAs includes ultraviolet (UV) lithography [6,7], thermal reflow [8,9,10], hot imprint [11,12] and ink-jet printing [13,14]. For example, in Taniguchi’s work [6], microlens arrays (MLAs) were obtained by curing a UV resin which was modified using ultrasonic vibration simultaneously. In another work, Ashraf [10] utilized the thermal reflow method to fabricate MLAs. The cured photoresist cylinder will be melted at a certain temperature and eventually turn into a hemispherical shape under the effect of surface tension. However, due to their high thermal expansion coefficient and low chemical and mechanical stability, polymer MLAs are prone to deformation and wear.

Glass MLAs have a comparatively higher transparency, chemical and mechanical stability and abrasion resistance; therefore, they can be applied in extreme and special environments. The conventional method for fabricating glass MLAs is femtosecond laser modification assisted etching [15,16,17]. In this method, the glass substrate is modified using a femtosecond laser in micro-regions at first, and then the structure is etched using an ion beam or a chemical solution to obtain MLAs. For instance, based on femtosecond laser modification and ion beam etching, Liu et al. [18] produced MLAs with a surface roughness of 2.5 nm and focal length of 60 to 100 μm in fused silica. Qin et al. [19] implemented MLAs by modifying fused silica with a femtosecond laser and processing with HF etching. The depth and radius of the modified area could be adjusted flexibly by controlling the laser energy and pulse delay. At last, MLAs with a numerical aperture in the range of 0.1 to 0.65 were obtained. In the above cases, the MLAs were concave-shaped when the femtosecond-laser-modified glass was removed from the substrate. Some scholars obtained convex glass MLAs by reverse-molding the concave MLAs with softened or molten glass. Li et al. [20] fabricated a concave microlens template with femtosecond laser modification assisted HF etching first. Then, by placing thermally softened As_2_Se_3_ glass onto the template, they finally obtained a 1600 rectangular and hexagonal convex MLA. Liu et al. [21] reported the fabrication of over 190,000 convex MLAs using reverse molding as concave compound eye templates, which were produced in sapphire using femtosecond laser modification assisted etching, and utilizing melted K9 glass. Definitely, the femtosecond laser modification assisted etching methods were suitable for high accuracy, low surface roughness and excellent homogeneity in glass MLA fabrication. However, these methods ask for point-by-point high-energy femtosecond laser processing of the material, which results in high cost and long fabricating times in high-throughput production. In addition, Shi [22] presented a work of fabricating germanium MLAs by utilizing reactive ion etching (RIE), which is capable of fabricating glass MLAs as well. However, RIE instruments are usually large and complex, the procedure is complicated and the fabrication usually costs a lot. In recent years, 3D printing techniques have been applied to printing SiO_2_-doped polymeric materials, which have shown a promising way of fabricating glass microstructures [23]. Kotz et al. [24,25] constructed a model for a microlens using SiO_2_-nanoparticle-doped photo-curable resin, and then the model was thermally treated to debind the polymerized resin. The remaining SiO_2_ nanoparticles in the model were sintered and eventually converted into fused silica glass. However, this kind of glass additive fabrication method has only been reported in the processing of larger-size structures. In addition, the sintering temperature reaches as high as 1300 °C, which requires high-standard sintering devices.

In this paper, we proposed a new method, namely UV-lithography-assisted sintering (UVLAS), to implement convex glass MLAs. In this method, low-melting-point micron glass particles (GPs) were doped in the photoresist (PR) and immobilized as large-scale micropillar structures via UV lithography. Subsequently, the polymerized PR in the micropillars was removed and the GPs were melted via a sintering process. Finally, convex glass MLAs with focal lengths ranging from 0.12 to 0.2 mm were obtained. This method is capable of high throughput and mass production of glass MLAs compared to femtosecond laser modification assisted etching. The fabrication system and procedure are much simpler and the MLA fabrication cost is much lower compared to techniques with large instruments such as RIE. Further, it could be applied to micrometer-scale fabrication, and is safer and cost-saving compared with the above-mentioned sintering method. Therefore, UVLAS is promising for the rapid and efficient preparation of convex glass MLAs.

## 2. Method and Materials

The sintering method of glass MLAs is a high-temperature version of fabricating polymer MLAs using thermal reflow, as in references [8,9,10]. The shaping of the MLAs mainly relies on the surface tension of the material in the liquid phase. Therefore, there are three factors to ensure the uniformity of lenses in MLAs. First, the glass particles need to be uniformly dispersed into the photoresist to ensure that each lens contains the same amount of glass particles. Second, the quartz glass substrate needs to be very clean before spin-coating the GP-doped PR, as well as after development, to avoid the morphological flaws caused by impurities. Third, the bubbles in the sintering should be well controlled by using appropriate sintering conditions.

**Figure 1:**
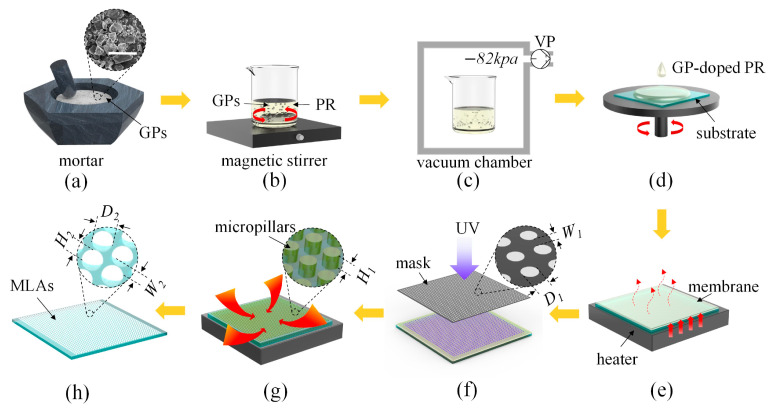
Schematic of fabricating MLAs using UVLAS. (**a**) Grinding; (**b**) stirring; (**c**) degassing; (**d**) spin coating; (**e**) baking; (**f**) UV lithography; (**g**) sintering; (**h**) shaping.

As shown in Figure 1, the procedure of UVLAS consists of eight stages. (1) Grinding: Low-melting-point micron GPs (D255, Anywhere New Materials Co., Ltd., Guangzhou, China) with an average particle size of 5 μm were, respectively, washed with acetone, isopropanol and pure water for 30 min to remove impurities. The GPs were dried and grinded with a mortar, and then finally sieved using a 2800 mesh standard sieve (shown in Figure 1a, the scale bar refers to 10 μm). Moreover, the compositions and the corresponding weight ratios of the GPs are summarized in Table 1. (2) Stirring: The sieved GPs were dispersed into the PR (SU-8 3005, Kayaku Advanced Materials Inc., Westborough, MA, USA) with a weight ratio of 45.5 wt% and stirred for 10 h at low speed with a magnetic stirrer (shown in Figure 1b). (3) Degassing: The GP-doped PR should be put into a vacuum chamber and degassed at −82 kpa for 2 h to remove the air bubbles (shown in Figure 1c). (4) Spin coating: The degassed GP-doped PR was spin-coated (2500 rpm for 30 s) on a quartz glass substrate and the thickness of the spin coating *H*_1_ was 13.5 μm (shown in Figure 1d). (5) Baking: The sample was placed onto a heater and baked at 95 °C for 10 min to solidify the GP-doped PR as a membrane (shown in Figure 1e). (6) UV lithography: The membrane was exposed using a UV lithography system (MDA-400LJ, Midas Systems Co., Ltd., Daejeon, Republic of Korea) 10 times (10 s for each exposure and 10 s as an interval) and the sample was baked for 10 min after exposure to sharpen the structure of the pattern after lithography (shown in Figure 1f). Then, the baked sample was processed using developer to obtain the micropillar structures. (7) Sintering: The micropillar structures were sintered in an atmosphere furnace (CQ-ZKQF18E, Luoyang Chunqing Furnace Industry Co., Ltd., Luoyang, China) (shown in Figure 1g). (8) Shaping: The microlenses were self-assembled under surface tension and then MLAs were produced (shown in Figure 1h).

## 3. Results and Discussion

The sintering of the samples was carried out in an atmospheric furnace and the sintering process includes four stages: preheating, debinding, melting and cooling. The debinding stage focused on removing the polymerized PR from the micropillar using thermal decomposition. Since non-optimized debinding conditions can cause bubbles and cracks, which in turn affect the performance of the microlens, prolonged heating was necessary to give the polymerized PR sufficient time to decompose [26]. At the melting stage, the remaining GPs after debinding were melted and spontaneously assembled as a microlens under the effect of the surface tension of the molten glass. Herein, by applying different processing temperatures and duration times in the sintering, the effect of processing temperature and duration time on the flaws in the MLAs was investigated. Then, the optimum sintering parameters suitable for low-melting-point GPs were obtained.

**Figure 2:**
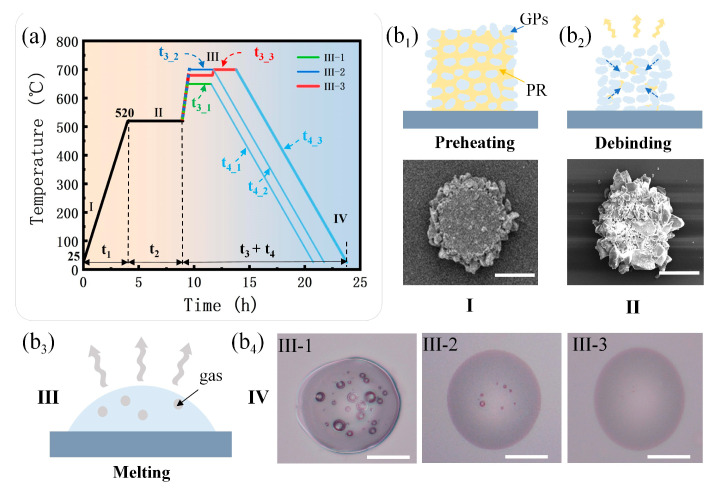
Sintering process of MLAs utilizing UVLAS. (**a**) Sintering scheme; (**b_1_–b_4_**) schematic diagrams of the microstructure and morphology changes in the MLAs at different stages; (**b_1_**) preheating; (**b_2_**) debinding; (**b_3_**) melting; (**b_4_**) cooling.

As shown in Figure 2a, the four sintering stages are labeled I to IV, and the microstructure and morphology changes in the samples corresponding to each stage are shown in Figure 2(b_1_–b_4_). Stage I is preheating: the temperature in the furnace was raised from room temperature to the debinding temperature at a rate of 2 °C/min. The corresponding lasting time was t_1_. Because the softening temperature of the GPs is 550 °C, the debinding temperature was set at 520 °C. The microstructures of the samples, i.e., the micropillars, at stage I consisted of the polymerized PR and GPs (Figure 2(b_1_)). Ⅱ is the debinding stage corresponding to the time period t_2_. The micropillars were debound at 520 °C for 6 h. At this stage, the polymerized PR in the micropillars was removed using thermal decomposition; meanwhile, the GPs were gathered inwardly, and the space between the GPs became smaller (Figure 2(b_2_)). III is the melting stage corresponding to the time period t_3_. At this stage, due to the furnace structure and heating method, the outer GPs melted first and wrapped the incompletely melted GPs of the inner side, thus preventing the escape of the gas, which remained between the GPs or was produced during the melting of the GPs. These phenomena resulted in the tiny bubbles inside the sintered structure (Figure 2(b_3_)). Herein, three different melting conditions (III-1, III-2, III-3) were used for comparative study. The melting temperature T_3_1_ and duration time t_3_1_ in melting condition III-1 were 650 °C and 2 h, respectively. In melting condition III-2, the melting temperature T_3_2_ was 700 °C and the duration time t_3_2_ was 2 h. The melting parameter corresponding to melting condition III-3 was 680 °C for 2 h, and then warmed up to 700 °C for 2 h. The optical images of the microlenses after cooling (stage IV) corresponding to the three sets of sintering conditions are shown in Figure 2(b_4_). At the cooling stage, the microstructures slowly cooled down from the temperature of stage III to room temperature at a rate of 1 °C/min to ensure that the structures were heated uniformly and avoid the generation of cracks on the surface. From left to right in Figure 2(b_4_), the illustration showing large and densely distributed bubbles in the structure corresponds to condition III-1. The microlens with fewer bubbles corresponds to the result of condition III-2, while the bubble-free microlens is obtained corresponding to III-3. The scale bars refer to 20 μm in Figure 2(b_4_).

It can be seen that increasing the melting temperature can reduce the viscosity of the molten glass, which can accelerate the rising speed of the submerged bubbles in the molten glass. Furthermore, the rise rate of the bubbles is proportional to the quadratic of the bubble radius, so bubbles with larger radii will have a higher rise rate [27,28,29]. Therefore, when the melting condition was changed from III-1 to III-2, the rising speed of the bubbles increased as the viscosity of the molten glass reduced with a higher temperature. Finally, bubbles with larger diameters were eliminated while small-diameter bubbles were left for the insufficient processing time of III-2. In case of condition III-3, all the bubbles were eliminated as the viscosity of the molten glass was low and the corresponding phase lasted long enough. In addition, a stepwise increase in temperature was adopted, which might lower the crack risk of the quartz glass substrate. In summary, it can be concluded that reducing the viscosity of the molten glass and appropriately extending the period of low viscosity are the key factors to eliminate bubbles and control the quality of MLAs.

**Figure 3:**
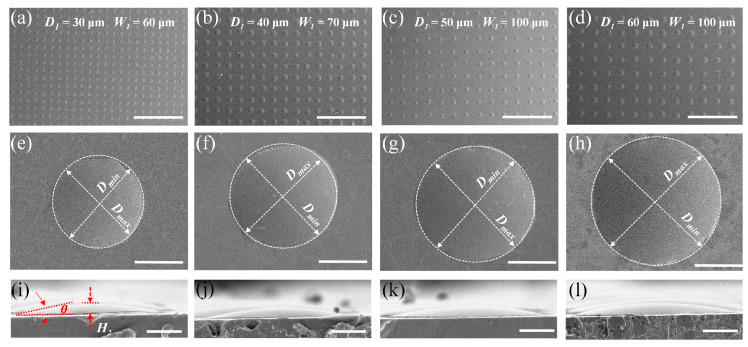
Fabrication results of MLAs with different lithography masks. (**a**) *D*_1_ = 30 μm, *W*_1_ = 60 μm; (**b**) *D*_1_ = 40 μm, *W*_1_ = 70 μm; (**c**) *D*_1_ = 50 μm, *W*_1_ = 100 μm; (**d**) *D*_1_ = 60 μm, *W*_1_ = 100 μm; (**e**–**h**) magnified view of individual microlenses, respectively, in (**a**–**d**); (**i**–**l**) height morphology of individual microlenses, respectively, in (**e**–**h**). The scale bars in (**a**–**d**), (**e**–**h**) and (**i**–**l**) are 500 μm, 20 μm and 10 μm, respectively.

**Figure 4:**
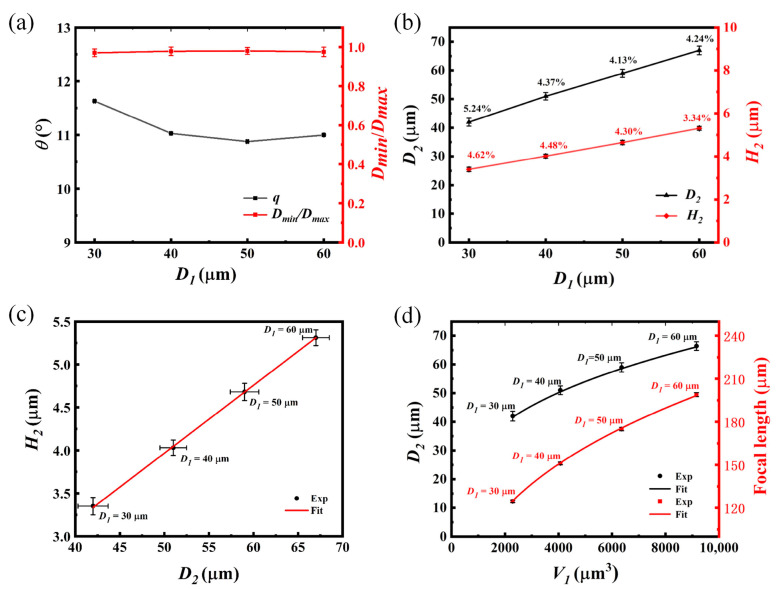
Analysis of MLA fabrication results based on UVLAS. (**a**) Contact angles and the morphological symmetry of MLAs fabricated with different lithography masks; (**b**) variation in microlens diameter *D*_2_ and height *H*_2_ with respect to *D*_1_ (The percentage indicates the error ranges of the fabrication results); (**c**) variation in the microlens height *H*_2_ in relation to microlens diameter *D*_2_; (**d**) variation in *D*_2_ in relation to *D*_1_, and the focal length f of the MLAs in relation to volume *V*_1_ of the GPs.

Based on the UVLAS method, circular lithography patterns with different diameters were applied to the fabrication of MLAs. The fabrication results are shown in Figure 3. In Figure 3a–d are the results of MLAs processed with different masks. The diameters of the circular patterns of the lithography mask, *D*_1_, are 30, 40, 50 and 60 μm, respectively. Different spacing *W*_1_ was adopted in these masks so the ultimate microlenses are different. It can be seen that the MLAs are uniformly round-shaped and evenly arranged. In addition, Figure 4b indicates that the error ranges of the prepared MLAs are 4.13% to 5.24% and 3.34% to 4.62% for the diameter *D*_2_ and height *H*_2_, respectively, which are too slight to influence the uniformity of MLAs both in shape and arrangement. Figure 3e–h show the zoomed-in images of the single microlenses, respectively, in Figure 3a–d. Let *D_min_* and *D_max_* be the minimum and maximum of the diameter of the microlens at the bottom contours. The ratio *D_min_*/*D_max_* could evaluate the morphological symmetry of the fabricated microlens. As the value of *D_min_*/*D_max_* is close to 1, the bottom profile of the microlens would be closer to an ideal circle. As shown in Figure 4a, the values of *D_min_*/*D_max_* for the microlenses obtained using the four sets of lithographing masks are all above 0.970; thus, the microlens profiles were complete and closed to an ideal circle.

Figure 3i–l show the height morphology of the above four sets of microlenses corresponding to Figure 3e–h. It can be found that the morphology of the microlenses was intact and smooth. The contact angle of the different-sized microlenses (as highlighted in Figure 3i) was measured to be 11° with a slight fluctuation (up to 0.6°) (shown in Figure 4a). This is because the contact angle is related to the properties of the molten glass and the quartz glass substrate itself. Despite the volume of the molten glass droplets, the contact angle is constant at a certain sintering temperature.

Figure 4 shows the analysis of the MLA fabrication results based on UVLAS. Let *D*_2_ be the diameter and *H*_2_ be the height of the fabricated microlens. The diameters *D*_2_ of the microlenses were larger than the *D*_1_ of the masks, and the increments were, respectively, 0.376, 0.265, 0.172 and 0.110, corresponding to *D*_1_ = 30, 40, 50 and 60 μm. The corresponding mean values of the microlens heights *H*_2_ were 3.30, 4.02, 4.65 and 5.31 μm, respectively. It seems abnormal that the molten glass spread wider as the lithographed capillary was smaller. However, from Figure 4b, it can be found that the diameter *D*_2_ and the height *H*_2_ linearly increased. Further, from Figure 4c, the ratio of *D*_2_ and *H*_2_ was approximate to a constant (0.079), and the tangent angle corresponding to *H*_2_ and the radius of the MLAs was maintained around 9° (quite close to the contact angle *θ*). These findings indicate that the spread of the microlens on the substrate is always determined by the surface tension of the molten glass. Regardless of the volumes of the microlens, the contact angle of each microlens should be same.

Subsequently, the variation in the diameter *D*_2_ and focal length f of the MLAs with respect to the volume *V*_1_ of the GPs in the micropillar before sintering is plotted and shown in Figure 4d. It is seen that the diameter *D*_2_ and the focal length f enlarged with an increase in *V*_1_. To further understand the mechanism of the changes, the following derivation was carried out. Assume *V*_1_ is the volume of the GPs in the micropillar structure before sintering, *V*_2_ is the volume of the microlens after sintering and f refers to the focal length of the microlens: they have the following expressions:

(1) $$V_{1}=\alpha \pi {\left(\frac{D_{1}}{2}\right)}^{2}H_{\begin{array}{l} 1 \end{array}}$$

(2) $$V_{2}=\frac{1}{6}\pi H_{2}\left[3{\left(\frac{D_{2}}{2}\right)}^{2}+H_{2}^{2}\right]$$

(3) $$f=\frac{D_{2}^{2}+4H_{2}^{2}}{8H_{2}\left(n-1\right)}$$

where *α* in Equation (1) is the ratio of the GPs to the volume of the micropillars, which can be calculated using the weight and density of GPs and PR, and the value of *α* is 0.240 in this paper. *n* in Equation (3) is the refractive index of the GPs, *n* = 1.54. 

In this paper, the loss of GPs in the process of sintering was very slight; thus, *V*_1_ was approximately equal to *V*_2_. Substituting *H*_2_/*D*_2_ = 0.079 into Equations (1)–(3), respectively, can derive the relationship of diameter *D*_2_ and the focal length f to *V*_1_. As shown in Figure 4d, the experimental results fit with the derived functions. Therefore, the diameter *D*_2_ and focal length f of the microlens after sintering could be calculated by knowing the volume *V*_1_ of the GPs before sintering. Similarly, this method could be applied to fabricating the MLAs with controllable diameter *D*_2_ and focal length f by adjusting the volume *V*_1_ when the *H*_2_/*D*_2_ is approximately a constant value.

**Figure 5:**
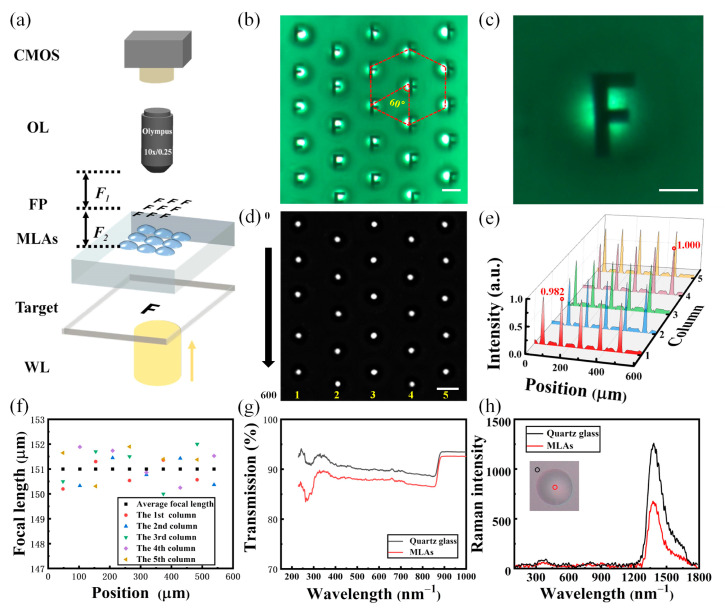
Imaging performance of MLAs using UVLAS. (**a**) Schematic diagram of the optical measuring system; (**b**) optical imaging utilizing MLAs; (**c**) magnified optical imaging of a single microlens; (**d**) focusing performance of MLAs; (**e**) focal length analysis of MLAs; (**f**) characterization of the normalized light intensity distribution of the MLAs; (**g**) ultraviolet–near-infrared transmission spectra of MLAs and quartz glass substrate; (**h**) Raman spectra of MLAs and quartz glass substrate. The scale bars in (**b**–**d**) refer to 50, 15 and 50 μm, respectively.

Finally, an imaging system as shown in Figure 5a was built to test the imaging performance of the MLAs. The system was constructed using a wide-field light source (WL), an imaging target, an objective lens and a CMOS camera. In the system, the focal plane (FP) of the objective lens was aligned overlapping with the FP of the microlens. MLAs arranged in hexagons and obtained using a mask with *D*_1_ = 40 μm and spacing *W*_1_ = 70 μm were utilized in the test. As shown in Figure 5b, when the target was illuminated with WL, the target letter “F” could be clearly observed as the array of “F” using the CMOS camera (MER-2000-19U3C-L, Daheng New Epoch Technology, Inc., Beijing, China). Figure 5c is a magnified optical image of a single microlens observed using a 40× objective lens. From Figure 5c, it can be seen that the target letter “F” is clear and undeformed. In Figure 5d, there are five columns of microfocus obtained by the MLAs in Figure 5b, and it can be seen that the spots are uniformly distributed in hexagons in the FP. The scale bars in Figure 5b–d refer to 50, 15 and 50 μm, respectively. The actual focal lengths of all the microlenses in Figure 5d are listed in Figure 5e. The average focal length of the microlenses was calculated based on Equation (3), which is 151 μm. The actual value of the lens focal length fluctuated around the mean value with a maximum error of 0.66% compared to the mean focal length. The clear and uniform images indicate that the focal planes of each microlens were essentially flush with each other. In addition, the normalized light field intensity at the focal cross-section of each column of microlenses in Figure 5d was plotted, and the results are shown in Figure 5f. The peaks of intensity were in the range of 0.982 to 1.000. The transmission spectra of the MLAs were plotted and compared with that of quartz glass in Figure 5g. The transmittance of MLAs has a bigger fluctuation in the range of 230–385 nm compared to the quartz glass substrate. The lowest transmittance (83.6%) and the maximum transmittance (92.7%) emerge at 264 nm and 890 nm, respectively. Meanwhile, the spectral transmittance of MLAs in the visible region is about 2% lower than that of quartz glass, while the spectral transmittance of the MLAs is comparable to that of quartz glass after 890 nm. At last, Raman spectra of the MLAs and quartz glass were plotted and compared in Figure 5h. It can be observed that the Raman spectrum shapes of MLAs and quartz glass are similar; the different intensity at 1380 nm can be attributed to the difference in SiO_2_ content in the two testing areas.

## 4. Conclusions

In this paper, a UV-lithography-assisted sintering method for convex glass microlens array fabrication was proposed and validated. It was found that lowering the viscosity of the molten glass and appropriately prolonging the duration of melting could eliminate bubbles in the microlenses. By utilizing four sets of different-sized masks, whose pattern diameters were, respectively, 30, 40, 50 and 60 μm, MLAs with average focal lengths of 122, 151, 175 and 201 μm were obtained successfully. The MLA morphology is symmetrically smooth, and the maximum error between the actual and theoretical focal length of the MLAs was 0.66%. It is found that both the diameter *D*_2_ and the focal length f of the microlens after sintering have a function relationship with the volume *V*_1_ of the GPs before sintering when the *H*_2_/*D*_2_ is approximately a constant value. Therefore, the size and focal length of the MLAs could be controllably adjusted by modifying the volume of the GPs. Finally, the imaging performance of the MLAs has been investigated. The results demonstrated the excellent imaging and focusing capabilities of the MLAs.

## Figures and Tables

**Table 1:** Information on the composition of GPs.

Composition	Na_2_O	MgO	Al_2_O_3_	SiO_2_	CaO	BaO	KF
**Weight ratio** **(wt%)**	18.43	2.06	4.47	55.17	8.12	5.68	6.07

## Data Availability

Not applicable.
